# Exposure to AT1 Receptor Autoantibodies during Pregnancy Increases Susceptibility of the Maternal Heart to Postpartum Ischemia-Reperfusion Injury in Rats

**DOI:** 10.3390/ijms150711495

**Published:** 2014-06-27

**Authors:** Hui-Ping Wang, Wen-Hui Zhang, Xiao-Fang Wang, Jin Zhu, Yan-Qian Zheng, Qin Xia, Jian-Ming Zhi

**Affiliations:** 1 Experimental Research Center, Shanghai Jiaotong University Affiliated First People’s Hospital, 650 New Songjiang Road, Shanghai 201620, China; 2 Department of Physiology, School of Medicine, Shanghai Jiaotong University, 280 Chongqing South Road, Shanghai 200025, China; E-Mails: jyzhangwj@shsmu.edu.cn (W.-H.Z.); wangxf95@gmail.com (X.-F.W.); jinz283@sohu.com (J.Z.); zyq-2-27@tom.com (Y.-Q.Z.); 3 Department of Pharmacy, Ruijin Hospital, School of Medicine, Shanghai Jiaotong University, 197 Ruijin Er Road, Shanghai 200025, China; E-Mail: xiaqin_@126.com

**Keywords:** angiotensin type 1 receptor autoantibodies, pregnancy, susceptibility, ischemia-reperfusion injury

## Abstract

Epidemiological studies have demonstrated that women with a history of preeclampsia have a two-fold increased risk of developing cardiovascular diseases in later life. It is not known whether or not this risk is associated with angiotensin II receptor type 1 autoantibody (AT1-AA), an agonist acting via activation of AT1 receptor (AT1R), which is believed to be involved in the pathogenesis of preeclampsia. The objective of the present study was to confirm the hypothesis that AT1-AA exposure during pregnancy may change the maternal cardiac structure and increase the susceptibility of the postpartum heart to ischemia/reperfusion injury (IRI). In the present study, we first established a preeclampsia rat model by intravenous injection of AT1-AA extracted from the plasma of rats immunized with AT1R, observed the susceptibility of the postpartum maternal heart to IRI at 16 weeks postpartum using the Langendorff preparation, and examined the cardiac structure using light and transmission electron microscopy. The modeled animals presented with symptoms very similar to the clinical symptoms of human preeclampsia during pregnancy, including hypertension and proteinuria. The left ventricular weight (LVW) and left ventricular mass index (LVMI) in AT1-AA treatment group were significantly increased as compared with those of the control group (*p* < 0.01), although there was no significant difference in final weight between the two groups. AT1-AA acting on AT1R not only induced myocardial cell hypertrophy, mitochondrial swelling, cristae disorganization and collagen accumulation in the interstitium but affected the left ventricular (LV) function and delayed recovery from IRI. In contrast, co-treatment with AT1-AA + losartan completely blocked AT1-AA-induced changes in cardiac structure and function. These data indicate that the presence of AT1-AA during pregnancy was strongly associated with the markers of LV geometry changes and remodeling, and increased the cardiac susceptibility to IRI in later life of postpartum maternal rats.

## 1. Introduction

Preeclampsia (PE) is a serious complication of pregnancy mainly characterized by hypertension, proteinuria and edema, with an incidence of about 10%. It threatens the health of both mother and fetus during pregnancy and is also the prime cause of premature birth and maternal/fetal death [1]. In addition, PE seriously affects the postpartum health of pregnant women. It is reported that women with a past history of PE have a significantly higher risk of developing hypertension [2], stroke [3], ischemic heart disease [4] and other cardiovascular diseases within 15 years postpartum as compared with normal women. Other studies have also reported that preeclamptic women have an increased risk of developing diabetes and chronic renal disease in their postpartum life, thus affecting their life expectancies [5,6,7,8].

Clinical studies have demonstrated that the cardiac structure and function undergo irreversible changes during the course of PE, constituting the main cause of disease progression and patient death. Even when pregnancy is terminated, the heart cannot completely restore its normal structure. Women with a history of PE have increased susceptibility to cardiac ischemic injury, larger post-ischemic infarct areas, more arrhythmias, and higher mortalities [3,4,5]. A current study by Heidrich, *et al.* [9] showed that most gynecologists and obstetricians know little about long-term hazards of PE on the cardiovascular system, and therefore are unable to give effective instructions concerning the prevention of postpartum development of cardiovascular diseases. Most preeclamptic women are completely unaware of their cardiac changes after giving birth, eventually leading to the occurrence of cardiovascular diseases.

Although the pathogenesis of PE remains elusive, imbalance of the renin angiotensin system (RAS) and the immune system is believed to be the main contributor [10,11]. More recent studies have shown that angiotensin II receptor type 1 autoantibody (AT1-AA) existing in the body and placenta tissue of preeclamptic women is closely associated with the pathogenesis of PE. The receptor target of AT1-AA is the second extracellular loop of AT1 receptor (AT1R), which plays an agonist-like effect. By activating AT1R, AT1-AA not only produces a physiopathologic cardiovascular effect but participates in the pathogenesis of PE by promoting the production of various substances that are closely associated with the pathogenesis of PE [11]. Our previous studies showed that AT1-AA could induce apoptosis of isolated myocardiocytes via the tumor necrosis factor-α (TNF-α) pathway [12], and that immunization of rats with artificially synthesized second extracellular loop (165–191) of AT1R to produce AT1-AA could induce apoptosis of rat myocardiocytes and change the cardiac structure and function [13]. Hubel *et al.* [14] showed that AT1-AA was detectable in women with a history of PE up to 18 months after delivery, and women with activating AT1-AA had significantly increased soluble fms-like tyrosine kinase-1, reduced free vascular endothelial growth factor. In our opinion, an abnormal elevation of AT1-AA during pregnancy is not only the main cause of PE but the cause of irreversible changes in cardiac structure and function in preeclamptic pregnant women and the risk factor contributing to the development of postpartum cardiovascular diseases. In the present study, we established a PE model by injecting AT1-AA to pregnant rats, and then observed the cardiac structure and function at 16 weeks postpartum, and tolerance of the heart to ischemia/reperfusion injury (IRI), in an attempt to analyze the cause of increased risk of developing cardiovascular diseases in postpartum women with a history of PE, and provide direct experimental clues for clinical treatment of preeclamptic heart disease and prevention of postpartum cardiovascular diseases.

## 2. Results and Discussion

### 2.1. Maternal and Fetal General Characteristics

**Figure 1:**
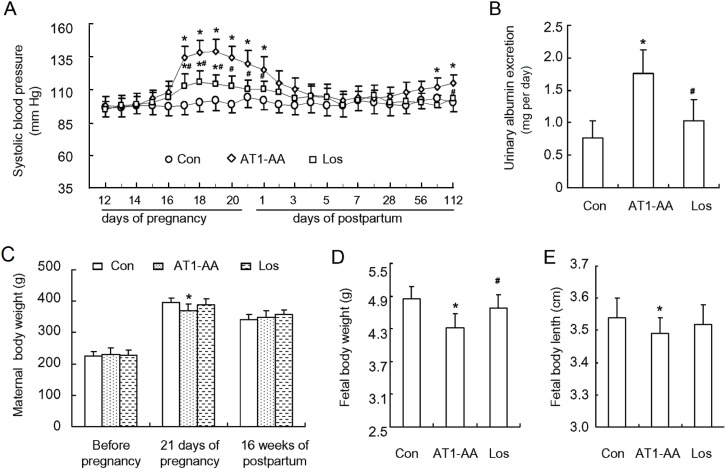
Affinity-purified AT1-AA (angiotensin II receptor type 1 autoantibody) induced an increase in SBP (systolic blood pressure) and proteinuria in pregnant rats. (**A**) SBP in pregnant rats after AT1-AA injection (1:640) at gestation day 13 in the presence or absence of losartan; (**B**) the ratio of urinary albumin/creatinine at gestation day 19; (**C**) maternal rat body weight (prior to, during and after pregnancy); (**D**) fetal rat body weight; and (**E**) fetal rat body length. Data represent the mean result from AT1-AA-injected rats, showing a significant increase in SBP and urinary protein. Con: control group; AT1-AA: AT1-AA group; Los: AT1-AA+losartan group. Data are expressed as means ± SD. (**A**–**C**): *n* = 6; (**D**,**E**): *n* = 32. * *p* < 0.05 *vs.* control group. ^#^ *p* < 0.05 *vs.* AT1-AA group.

There was no significant difference in body weight between the three groups before pregnancy. The mean body weight of the rats in AT1-AA group was slightly lower than that in control group one day before delivery, while there was no significant difference between AT1-AA + losartan group and control group (Table 2). There was no significant difference in systolic blood pressure (SBP) between the three groups before pregnancy (Figure 1A). SBP in AT1-AA group began rising at gestation day 16 (3 days after antibody injection), reached the peak (146 ± 12 mmHg) at gestation day 19, then declined gradually, and restored to normal at day 3 after delivery. However, SBP began rising gradually 12 weeks after delivery. Compared with control group, SBP in AT1-AA + losartan group was also elevated but the extent of SBP elevation was not so much as compared with AT1-AA group (Figure 1A). In addition, SBP in AT1-AA + losartan group was not elevated after delivery. At the same time, the rats of AT1-AA group displayed a significantly raised urinary protein excretion (Figure 1B). The gestational period of the three groups was between 21 and 22 days, showing no significant difference. Each rat bore 9–15 fetal rats, and there was no significant difference in the number of newborn rats between the three groups. However, the body weight and length of the newborn rats in AT1-AA group were significantly lower and shorter than those of control group. There was no significant difference in these parameters between AT1-AA + losartan and control groups (Figure 1D,E). There was no significant difference in body weight between the three groups at 16 weeks postpartum.

### 2.2. LV (Left Ventricular) Function and Post-Ischemic Recovery

**Figure 2:**
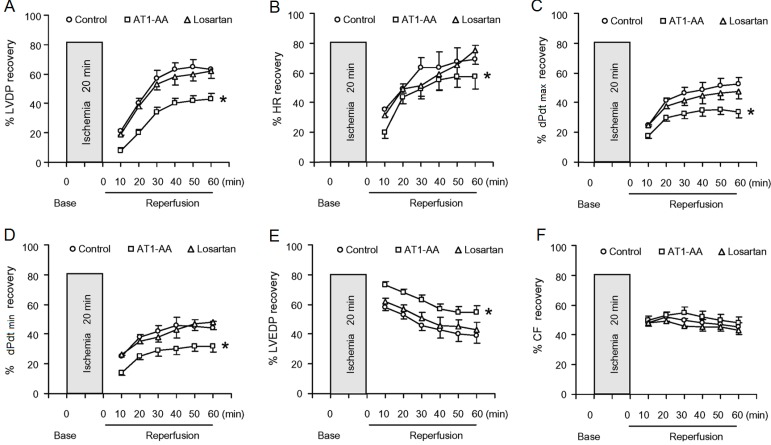
Effect of AT1-AA exposure on post-ischemic recovery of LV function (**A**–**E**) and coronary flow (**F**). Hearts obtained from the maternal rats exposed to either normal saline or AT1-AA (titer 1:640 0.1 mL/kg) at gestation days 13 and 14 were subjected to 20-min ischemia and 60-min reperfusion in the Langendorff preparation. LVDP: left ventricular developed pressure; HR: heart rate; dP/dt_max_ or dP/dt_min_: the maximal rates of pressure rise or fall; LVEDP: left ventricular end diastolic pressure; CF: coronary flow; Con: control group; AT1-AA: AT1-AA group; Los: AT1-AA + losartan group. * *p* < 0.05 compared with control group for the entire curve. *n* = 6 per group.

LV function and postischemic recovery using the Langendorff perfusion method. LV function was assessed in the isolated heart of the maternal rat exposed to either normal saline, AT1-AA, or AT1-AA + losartan at gestation day 13. The baseline left ventricular developed pressure (LVDP) and HR (heart rate) in AT1-AA group were slightly higher than those in control group, but there were no significant differences in left ventricular end-diastolic pressure (LVEDP), maximal rates of pressure rise or fall (dP/dt_max_ or dP/dt_min_) and coronary flow at baseline levels. Meanwhile, the hemodynamic parameters of cardiac function of control group and AT1-AA + losartan group were not significantly different (Table 1). Figure 2 shows the effect of 20-min ischemia followed by 60-min reperfusion on LV function in the three groups. Cardiac contractility in rats gradually slowed down, or even completely stopped during 20-min ischemia. Although the reduced cardiac contractility gradually recovered following reperfusion, but had not complete recovery to the baseline value even 40 min after reperfusion. Compared with control group, there were significant decreases in post-ischemic recovery of the LVDP, and HR and +dp/dt_max_ in AT1-AA-treated group was delayed significantly, However, postischemic recovery of coronary flow was no significant difference between control group and AT1-AA-treated group. Ischemia reperfusion (IR) caused an injury to the heart and resulted in elevated LVEDP during reperfusion in isolated rat hearts. However, there was a significant increase in the IR-induced elevation of LVEDP in the AT1-AA-treated heart, as compared with the control heart (Table 1). The functional parameters in AT1-AA + losartan group were significantly improved as compared with AT1-AA group.

**Table 1:** Pre-ischemic LV (left ventricular) function parameters.

Parameter	Control	AT1-AA	AT1-AA + Losartan
LVDP (mmHg)	116.6 ± 5.7	124.8 ± 6.2	118.2 ± 5.9
HR (beats /min)	309.2 ± 10.3	334.6 ± 13.2 *	304.2 ± 12.3 ^#^
LVEDP (mmHg)	5.71 ± 0.83	5.81 ± 0.86	5.74 ± 0.72
dp/dt_min_ (mmHg/s)	2483 ± 106	2410 ± 156	2380 ± 146
dp/dt_max_ (mmHg/s)	3985 ± 519	4120 ± 634	3836 ± 533
Coronary flow (mL/min)	8.07 ± 0.68	7.83 ± 0.67	7.92 ± 0.61

LVDP, left ventricular developed pressure; HR, heart rate; LVEDP, left ventricular end-diastolic pressure; * *p* < 0.05 compared with control; ^#^ *p* < 0.05 compared with AT1-AA group. Data are mean ± SD (*n* = 6).

### 2.3. Histopathological Change of the Myocardium

**Figure 3:**
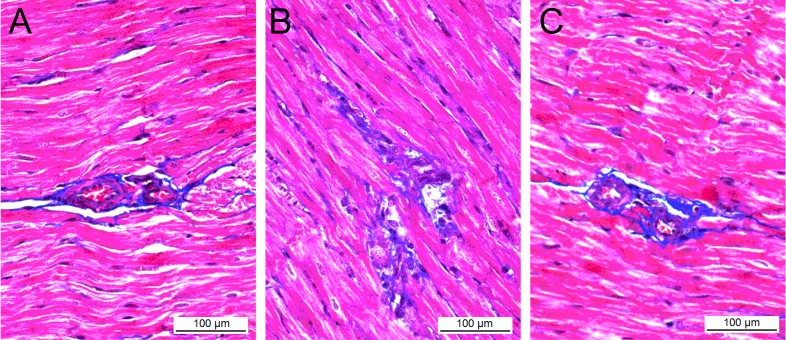
Masson’s trichrome stain of the heart in control group (**A**), AT1-AA group (**B**), and AT1-AA + losartan group (**C**). Where blue = fibrous collagen, and red = cardiomyocytes. (**A**) The comparative fibrosis of control rats (**B**) AT1-AA treated rats showing an increased amount of collagen deposition compared with control rats (*p* < 0.05); and (**C**) Less collagen accumulation in the heart of AT1-AA + losartan treated rats, compared with AT1-AA group (*p* < 0.05); representative histological sections.

Although no significant difference in body weight was observed between the three groups at 16 weeks postpartum, the absolute and relative left ventricular weight (LVW) and LVW/BW (body weight) in AT1-AA group were significantly higher than those in control group (Table 2). The histological changes of the myocardial tissue were observed via light microscopy after Masson staining. The myocardial cells in control group were oval or short spindled, and arranged in neat dense rows with smaller intracellular spaces and tightly stained uniform nuclei with round and clear nucleoli, and no significantly abnormal change in the cellular structure was seen (Figure 3A). The mean diameter of myocardial cells was 14.48 ± 2.79 μm, and the mean cross-sectional area (CSA) was 187.7 ± 32.9 μm^2^. In AT1-AA group, the number of myocardial nuclei per microscopic field was significantly reduced as compared with control group (728.6 ± 69.2 *vs.* 962.7 ± 67.4, *p* < 0.05), where cells were deranged and swollen, the nuclei were deeply stained, and cytolymph and necrosis were observed in the myocardial tissue (Figure 3B). 

**Table 2:** Heart weight index and morphometric analysis of LV sarcomeres and mitochondria of rats. LVW: left ventricular weight; BW: body weight.

Parameter	Control	AT1-AA	AT1-AA + Losartan
BW (g)	369 ± 11	358 ± 12	374 ± 14
LVW (mg)	689 ± 12	798 ± 14 *	708 ± 15 ^#^
LVW/BW (mg/g)	1.87 ± 0.08	2.23±0.12 *	1.94±0.09 ^#^
Infarct size (%)	17.6 ± 1.2	28.4 ± 1.8 *	19.7 ± 1.6 ^#^
Cardiomyocyte diameter (μm)	14.48 ± 2.79	18.13 ± 4.27	15.13 ± 3.12
Cross-sectional area (μm^2^)	187.7 ± 32.9	287.6 ± 59.4 *	201.2 ± 40.1 ^#^
Myocardial nuclei number	962.7 ± 67.4	728.6 ± 69.2 *	947.6 ± 74.3 ^#^

*n* = 6, * *p* < 0.05 *vs.* control group; ^#^ *p* < 0.05 *vs.* AT1-AA group.

The CSA of myocardial cells in AT1-AA group was 287.6 ± 59.4 μm^2^, which were significantly higher than those of control group (*p* < 0.01). No significant damage to the myocardial structure was observed in AT1-AA + losartan group, the mean diameter and CSA of myocardial cells compared with the control group did not differ significantly (*p* > 0.05) (Table 2).

### 2.4. Effects of AT1-AA (Angiotensin II Receptor Type 1 Autoantibody) on Myocardial Fibrosis

Myocardial fibrosis is related to excessive collagen synthesis, and hydroxyproline measurement can accurately reflect the amount of collagen in the tissue, by which we can indirectly understand the extent of myocardial fibrosis. The content of hydroxyproline in the myocardium of AT1-AA group was significantly higher than that in control group (*p* < 0.01), and there was less collagen accumulation in AT1-AA + losartan group compared with AT1-AA group, but there is no statistically significant difference (Table 3).

**Table 3:** Effects of AT1-AA on interstitial collagen volume fraction and peri-vascular collagen area-to-luminal area ratio of the LV.

Parameter	Control	AT1-AA	AT1-AA + Losartan
hydroxyproline content (mg/g)	2.14 ± 0.51	2.98 ± 0.76 *	2.47 ± 0.54
collagen content (mg/g)	15.82 ± 2.46	22.55 ± 4.23 *	18.47 ± 2.59 ^#^
collagen volume fraction (%)	3.72 ± 0.48	14.22 ± 1.54 *	5.79 ± 0.64 ^#^
perivascular collagen area-to-luminal area ratio (%)	5.76 ± 0.94	16.14 ± 2.12 *	8.11 ± 1.13 ^#^

Data are mean ± SD (*n* = 6). * *p* < 0.05 *vs.* control group; ^#^ *p* < 0.05 *vs.* AT1-AA group.

Cardiac fibrosis was observed in AT1-AA group, presenting as a diffuse, small, patchy and non-uniform pattern, where the collagen network structure in the interstitial and peri-vascular areas was destroyed and disorganized (Figure 3B). When compared with control group, the collagen volume fraction and perivascular collagen area/luminal area in AT1-AA group were markedly increased (*p* < 0.01 and *p* < 0.05, respectively). However, the collagen volume fraction and perivascular collagen area/luminal area in AT1-AA + losartan group were significantly lower than those in AT1-AA (*p* < 0.01 and *p* < 0.05, respectively), suggesting that Iosartan improved the AT1-AA-induced myocardial fibrosis (Figure 3C).

### 2.5. Ultrastructural Changes of the Myocardium

**Figure 4:**
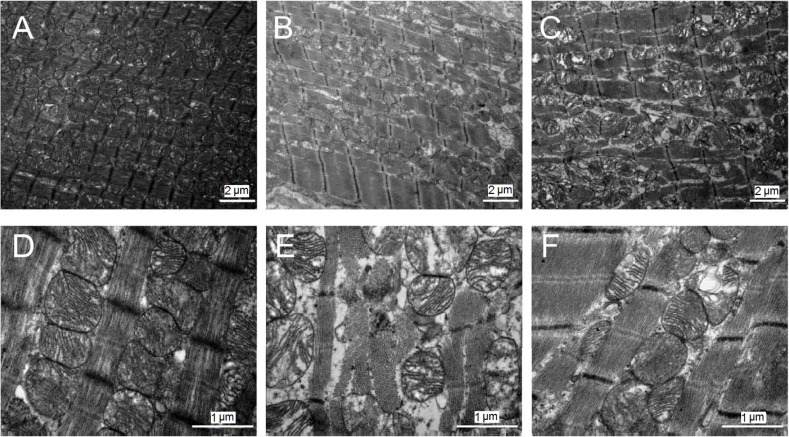
Transmission electron microscopy of myocardium after 20 min of ischemia followed by 60 min of reperfusion in control group (**A**,**D**), AT1-AA group (**B**,**E**), and AT1-AA + losartan group (**C**,**F**). (**A**,**D**) Normal nuclei and mitochondria in control group; (**B**,**E**) The mitochondria are swollen, partly disrupted, and contain flocculent densities; (**C**,**F**) Sarcomeres are moderately relaxed and mitochondria are swollen in AT1-AA + losartan group. Original magnification (**A**,**B**) 8400×; (**C**–**F**) 22,500×.

Microscopic observation on the LV ultrastructure in control group showed that myocardial fibers were arranged in neat rows, with bright bands, dark bands, Z lines and clear intercalated discs. The mean length of sarcomeres was 1.63 ± 0.07 μm. Mitochondria between the myocardial fibers were mostly oval in a beaded arrangement with clear lamellae (Figure 4A,D). In AT1-AA group, myocardial cells were wrinkled, myofibers were ruptured, and sarcomeres were blurred, with dissolution of sarcomeric organization and disruption of the intercalated disc. The length of sarcomeres averaged about 1.85 ± 0.11 μm. The number of mitochondria was decreased, and mitochondria were densely arranged. The cytoplasm was concentrated with more vacuoles inside. The mitochondrial ridge and inner-membrane fusion disappeared partially or completely (Figure 4B,E). In AT1-AA + losartan group, myocardial fibers were neatly arranged with clear intercalated discs and clear sarcomeres, the length of which averaged 1.68 ± 0.08 μm; the number of mitochondria gradually increased (Figure 4C,F), (Table 4).

**Table 4:** Morphometric analysis of rat LV sarcomeres and mitochondria.

Parameter	Control	AT1-AA	AT1-AA + Losartan
Sarcomere width (μm)	1.63 ± 0.07	1.85 ± 0.11 *	1.68 ± 0.08 ^#^
Sarcomere length (μm)	0.47 ± 0.06	0.74 ± 0.08 *	0.52 ± 0.06 ^#^
Volume density (%)	33.41 ± 3.62	25.22 ± 4.21 *	38.75 ± 3.66 ^#^
Numerical density (/μm^3^)	0.89 ± 0.11	0.75 ± 0.16	0.92 ± 0.15
Mean volume (μm^3^)	0.67 ± 0.08	1.02 ± 0.08 *	0.73 ± 0.08 ^#^

Data are mean ± SD (*n* = 6). * *p* < 0.05 *vs.* control group; ^#^ *p* < 0.05 *vs.* AT1-AA group.

### 2.6. Discussion

The volume of blood increases by more than 30% during pregnancy because of the establishment of fetal placental circulation and change of the endocrine system. In addition, enlargement of the uterus in the late stage of pregnancy pushes the diaphragm upward, causing upward and left anterior displacement of the heart, resulting in mild twisting of the major cardiac vessels, which further increases the burden of the maternal heart. The cardiac structure and function of pregnant women would be seriously affected when complications of hypertension, PE and diabetes occur. PE (eclampsia) constitutes the greatest threat to the heart of pregnant women both in developing and developed countries, and is also generally recognized as the main cause of premature birth, fetal and maternal death [1]. The study by Melchiorre *et al.* [15] study demonstrate that preterm PE is strongly associated with the persistence of LV dysfunction/hypertrophy at one year postpartum follow-up and the risk of development of essential hypertension within two years. As the pathogenesis of PE remains elusive, there is no effective means for the prevention and treatment of the condition at present. 

Ample studies have demonstrated that ventricular remodeling is closely associated with the abnormal RAS. Angiotensin II is the main component of the RAS. It induces hypertrophy, apoptosis and fibrosis of myocardial cells and causes other pathological changes, resulting in ventricular remodeling. AT1R inhibitors can effectively prevent or even reverse hypertrophy and fibrosis of myocardial cells, thus improving the cardiac function [16]. It is interesting to find that RAS components in the plasma of normal pregnant women are elevated significantly, while angiotensinogen, plasma renin activity and Ang II level in the plasma of preeclamptic pregnant women are significantly lower than those in normal pregnant women [9]. AT1-AA is a newly discovered immunoglobulin G (IgG) in the plasma of preeclamptic pregnant women. It can act on the second extracellular loop of AT1R to produce an Ang II agonist-like effect and pathologic impairment. Siddiqui *et al.* [17] found that plasma AT1-AA levels were elevated markedly in more than 95% of preeclamptic patients. Wenzel *et al.* [18] reported that AT1-AA could increase the sensitivity of pregnant rats to Ang II Combination use of AT1-AA and Ang II induced hypertension, proteinuria, intrauterine growth retardation and arteriolosclerosis in the uteroplacental unit, but use of Ang II alone did not induce these symptoms. Ample studies have demonstrated that AT1-AA participates in the pathogenesis of preeclampsia by inducing the expression of multiple cytokines closely associated with the onset of the disease [10,19].

Our previous studies showed that AT1-AA could induce hypertrophy and apoptosis of isolated myocardial cells in lactating rats [13], and antibodies produced by immunization of rats with the second extracellular loop of AT1R could induce apoptosis and hypertrophy of myocardial cells and remodeling of the myocardium, leading to hypertension [13]. The result of the presents study showed that pregnant rats injected with AT1-AA at gestation day 13 developed hypertension, and at gestational day 18 developed proteinuria, and the body weight and length of the newborn rats were lower and shorter. These manifestations are very similar to the symptoms of human preeclampsia, indicating that AT1-AA could also induce the occurrence of preeclampsia. At 16 weeks postpartum, the LVW and LVWI in the rats of AT1-AA group increased significantly; the CSA of myocardial cells was increased, and there were large amounts of collagen fiber deposition around the myocardial interstitial and vessels, indicating that the abnormally expressed AT1-AA in the plasma of preeclamptic women could lead to cardiac remodeling. This damage to myocardial cells was at the expense of increasing the size of myocardial cells, thus decreasing the functional reserve of the heart, and therefore the cardiac function can only meet the requirements of human resting levels. Under the condition of myocardial ischemia, the cardiac pumping function becomes insufficient. In addition, re-arrangement of myocardial cells is likely to trigger arrhythmia. As ventricular remodeling is a progressive process, LV dilatation, dysfunction and heart failure are prone to occur without effective prevention and treatment [20]. Studies [21] have shown that a hypertrophic heart has poor tolerance to ischemia, and is slow or unable to restore its function completely. It was observed in our study that the rat LVW, LVWI, CSA, collagen concentration and collagen area around blood vessels were all markedly decreased in AT1-AA + losartan group, and these changes were significantly different from those in rats treated with AT1-AA group (*p* < 0.05 or *p* < 0.01), indicating that losartan could delay or block the occurrence of AT1-AA induced myocardial hypertrophy and elevation of the collagen content in myocardial interstitial cells, thus attenuating or improving ventricular remodeling. Therefore, we postulate that AT1-AA existing in the plasma of pregnant women could induce cellular hyperplasia, which may be an important mechanism underlying the increased susceptibility of the postpartum heart to IRI. 

Histological study of the rat heart using light microscopy showed spotty and patchy necroses in myocardial cells in the rats of AT1-AA group. These necrotic lesions were mainly located under the endocardium, and partly across the full length of the ventricular muscular wall. They had been replaced by the proliferative fibrous tissue. Transmission electron microscopic examination of the myocardial ultrastructure showed that the myocardial cells had undergone degenerative, necrotic and hyperplasia-like changes with edematous interstitial and large amounts of collagen; part of the myofilaments were ruptured, and mitochondria were swollen without observing crests and with vacuolar degeneration observed. After the use of losartan, the ultrastructure of the myocardial cells was improved significantly as compared with that of the AT1-AA group. The structure of myocardial cells is the footstone of cardiac function. Damage to myocardial cells will decrease the cardiac pumping function, resulting in various cardiac diseases. Our previous study demonstrated that AT1-AA could enter the fetal rat via the placenta and induce the generation of large amounts of TNF-α, thus promoting apoptosis of myocardial cells of the fetal rat and thus increasing the susceptibility of the heart of these offspring to IRI 10 weeks postpartum. Xia *et al.* [10,11] found that the pathogenesis of AT1-AA-induced preeclamsia was closed associated with TNF-α. They found that the TNF-α concentration was increased markedly in the plasma of AT1-AA treated animals, and the use of TNF-α inhibitors could effectively attenuate AT1-AA-induced hypertension, preeclampsia and placental apoptosis. The result of the present study showed that SBP and HR were increased in AT1-AA group 16 weeks postpartum. The *in vitro* cardiac perfusion experiment showed that the LV function restored more slowly after myocardial ischemia, and the myocardial infarct area was also increased significantly, which may be due to the AT1-AA-induced generation of TNF-α, causing apoptosis of myocardial cells. 

The NLRP3 (nucleotide-binding domain leucine-rich repeat containing family, pyrin domain containing 3) inflammasome complex, assembled in response to microbial components or endogenous danger signals, triggers caspase-1-mediated maturation and secretion of IL-1β and IL-18 processing, a key step in the innate immune response [22]. Several studies [23,24] have shown that cardiac myocytes and cardiac fibroblasts express NLRP3, which is involved in the pathogenesis of myocardial ischemia-reperfusion injury, while the NLRP3 inflammasome is up-regulated in myocardial fibroblasts after myocardial infarction, and may be a significant contributor to infarct size development during IR. NLRP3 agonists that have been tested trigger the production of reactive oxygen species (ROS) [25]. ROS production results in NLRP3 inflammasome activation through release of the ROS-sensitive NLRP3 ligand thioredoxin-interacting protein from its inhibitor thioredoxin [26]. Parrish *et al.* [27] suggest that AT1-AA through activation of NADPH oxidase could contribute to ROS production and inflammatory responses in PE. Therefore, our future plan is to further study the role and participation of NLRP3 inflammasome in AT1-AA-induced increases in cardiac susceptibility to IRI.

## 3. Experimental Section

### 3.1. Animals

SPF Wistar rats (Experimental Animal Center of Shanghai Jiaotong University School of Medicine, Shanghai, China) were fed normal rat chow and tap water *ad libitum* with a 12:12 h light-dark cycle (lights on at 19:00 h) at a constant ambient temperature (23 ± 2 °C) and humidity (60% ± 5%). All experimental protocols were approved by the Experimental Animal Care and Use Committee of Shanghai Jiao Tong University School of Medicine.

### 3.2. AT1-AA Detection and Affinity Purification

AT1-AA extracted from the plasma of patients with PE, which was diagnosed according to the criteria of the International Society for the Study of Hypertension in Pregnancy. The study was approved by the Ethics Committee of Shanghai Sixth People’s Hospital affiliated to Shanghai Jiao Tong University, and written informed consent forms were signed by all subjects. Serum antibody titer and purified antibody dilution were measured by using the enzyme-linked immunosorbent assay (ELISA) method described previously [15]. AT1-AA were purified by MAb Trap Kit (Amersham, Piscataway, NJ, USA) according to the manufacturer’s instructions. Before use, the purified antibody was diluted with phosphate-buffered saline (3.2. mM Na_2_HPO_4_, 0.5 mM KH_2_PO_4_, 1.3 mM KCl, 135 mM NaCl, pH 7.4) to an antibody titer of greater than 1:640 by ELISA detected. 

### 3.3. Establishment of the PE (Preeclampsia) Rat Model

A PE rat model was developed by using AT1-AA as previously described [14]. Virgin female Wistar rats (220~240 g) were mated overnight with sexually experienced males, and pregnancy was confirmed by the presence of a vaginal plug of semen in the mating cages on the following morning (gestation day 1). Eighteen rats were equally randomized into a control group, an AT1-AA treated group (AT1-AA group), and an AT1-AA + losartan treated group (AT1-AA + losartan group). AT1-AA (100 μL PBS, titer > 1:640) was administered to the pregnant rats via tail vein injection at gestation day 13 and again at day 14 (term = 22–23 days). The AT1-AA + losartan group was treated with losartan at 10 mg/kg once a day by gavage from gestation day 14 to 18. IgG of equal protein concentration from normotensive pregnant women was used as a control. At gestation day 19, the pregnant rats were placed in individual metabolism cages for 24 h to collect urine for measurement of urinary albumin concentration and the urinary albumin/creatinine ratio. SBP was measured before, during and after gestation day 13. Blood pressure was recorded in unanesthetized rats using the tail-cuff method. The pups were counted and weighed, and the body length (from the top of head to the tail base) was measured within the first 24 h after birth. After delivery, the maternal rats of each group were conventionally reared until use for experiments.

### 3.4. Subjection of the Perfused Rat Heart to Ischemia-Reperfusion

At 16 weeks after giving birth, the maternal rats were systemically heparinized (500 U, *i.p.*) and anesthetized with sodium pentobarbital (60 mg/kg, *i.p.*). The heart was excised rapidly and retrogradely perfused via the aorta in a modified Langendorff apparatus under constant pressure (60 mmHg) with gassed (95% O_2_, 5% CO_2_) Kreb’s solution at 37 °C as previously described [14]. A latex balloon filled with Kreb’s solution was inserted into the left ventricle (LV) to record of LV hemodynamic parameters by a digital acquisition and analysis system (PowerLab/4SP, ADInstruments, Pty. Ltd., Castle Hill, Australia). In each case, LV end diastolic pressure (LVEDP) was initially set at approximately 5 mmHg by adjusting the volume of fluid in the balloon. The indices of hemodynamic parameters including heart rate (HR), LV developed pressure (LVDP), and the maximal rates of pressure rise or fall (dP/dt_max_ or dP/dt_min_) were recorded. Pulmonary artery effluent was collected as an index of coronary flow (CF). After the baseline recording, the heart was subjected to 20-min global ischemia by stopping the reperfusion, followed by 60-min reperfusion. 

### 3.5. Sample Collection

At the end of reperfusion, a piece of myocardial tissue was cut from the apex for transmission electron microscopic study. Then, the right ventricle (RV) and both atria were trimmed from the LV. After being weighed, the LV was cut into three transverse sections: apex, middle ring (3 mm), and base as previously reported [27]. The apex and base sections were freeze-clamped in liquid nitrogen and stored at −80 °C for biochemical testing until analysis. The middle section was fixed in 10% buffered formalin at 4 °C for histological examination. Cardiac hypertrophy was assessed by measuring the LV weight (LVW, in mg) and the ratio of LVW to BW (in g) (LVW/BW, in mg/g). 

### 3.6. Measurement of the Myocardial Infarct Size and Ventricular Remodeling

The middle part of the heart was formalin-fixed, paraffin-embedded, sliced into 5-µm sections at 300-μm intervals, mounted on glass slides, and stained with Masson trichrome stain to discriminate the nucleus (blue-black), collagen and cytoplasm (blue), and muscle fibers (red). The boundary lengths of the infarcted and non-infarcted endocardial and epicardial surfaces were traced with a planimeter digital image analyzer. The infarct size (fraction of the infarcted LV) was calculated as the average of all slices and expressed as the percentage of the length of circumference. The collagen volume fraction and perivascular collagen area/luminal area were analyzed by quantitative morphometry with automated image analysis (Image-Pro Plus, version 5.0; Media Cybernatics, Houston, TX, USA). The cross-sectional area (CSA) of myocardial cells was determined using the method as described previously [12]. The size of myocardial cells in each group was evaluated by measuring the CSA of myocardial cells. The mean myocardial cell area was calculated by measuring 100 cells from the stained sections. LV cavity area (area enclosed by LV endocardial circumference) was used as an index of LV dilatation.

### 3.7. Observation of the Myocardial Ultrastructure

Myocardial fibers were fixed in 2.5% glutaraldehyde, post-fixed in osmium tetroxide, embedded in epoxy resin, sliced into ultrathin sections, and stained with saturated uranyl acetate and lead citrate for electron microscopic examination. For each subject, five micrographs were sampled to obtain measurements of sarcomere width (short axis) and length (long axis), mitochondrial volume density, numerical density, and mean volume were measured as described previously. Measurements of sarcomere width were performed on eight sarcomeres per photomicrograph [28]. 

### 3.8. Quantification of LV Collagen

LV collagen was determined by estimating the content of hydroxyproline, an amino acid characteristic of collagen (100 g collagen containing approximately 13.4 g hydroxyproline). The content of hydroxyproline in the myocardial tissue was assayed using a commercial kit (Nanjing Jiancheng Bioengineering Institute, Nanjing, China) according to the manufacturer’s instructions. Briefly, the homogenate of an approximately 5.0 mg LV sample was centrifuged at 1000× *g* for 10 min. The absorbance of the supernatant containing hydroxyproline was measured by spectrophotometry at a wave length of 550 nm. The gross collagen protein content of a specimen was then estimated using the equation: Gross collagen content = Hydroxyproline content/13.4%.

### 3.9. Statistical Analysis

Data analyses were performed using SPSS software version 14.0 (SPSS Inc., Chicago, IL, USA). Results were reported as mean ± SD. Two-way repeated-measures mixed model ANOVAs (analysis of variance) were used with *post-hoc* tests to compare cardiac function in baseline and IR experiments between each treatment group at each pressure level and/or time-point. Results were considered statistically significant at *p* < 0.05. 

## 4. Conclusions

Women with a history of preeclampsia are susceptible to the postpartum development of cardiovascular diseases due to AT1-AA-induced ventricular remodeling, increased susceptibility of the heart to ischemic injury, increased myocardial infarct size, and decreased ability of the heart to recover the LV function after ischemia. The exact reason for the increased susceptibility of the myocardium to ischemia after giving birth in women with a history of preeclampsia remains unclear. However, TNF-α, AT1-AA and other pathogenic factors may play important roles in increasing the postpartum susceptibility of the heart to IRI by producing persistent or even lifetime damage to the heart via a series of complex signaling pathways. 
